# Observational Learning: Tell Beginners What They Are about to Watch and They Will Learn Better

**DOI:** 10.3389/fpsyg.2016.00051

**Published:** 2016-01-29

**Authors:** Mathieu Andrieux, Luc Proteau

**Affiliations:** Département de kinésiologie, Université de MontréalMontréal, QC, Canada

**Keywords:** action observation network, motor learning, knowledge of results, feedback, feedforward, relative timing

## Abstract

Observation aids motor skill learning. When multiple models or different levels of performance are observed, does learning improve when the observer is informed of the performance quality prior to each observation trial or after each trial? We used a knock-down barrier task and asked participants to learn a new relative timing pattern that differed from that naturally emerging from the task constraints (Blandin et al., 1999). Following a physical execution pre-test, the participants observed two models demonstrating different levels of performance and were either informed of this performance prior to or after each observation trial. The results of the physical execution retention tests of the two experiments reported in the present study indicated that informing the observers of the demonstration quality they were about to see aided learning more than when this information was provided after each observation trial. Our results suggest that providing advanced information concerning the quality of the observation may help participants detect errors in the model's performance, which is something that novice participants have difficulty doing, and then learn from these observations.

## Introduction

You are an avid golfer and you want to learn a new shot. How would you proceed? There is a fair chance that you will observe someone (live, on video, on Youtube, etc.) who knows how to perform this shot, and you will try to understand what to do and how to do it. Research clearly indicates that this learning strategy is successful because observation has been shown to promote the learning of a wide variety of motor skills (see McCullagh et al., 1989; Hodges et al., 2007; Vogt and Thomaschke, 2007; Ste-Marie et al., 2012; Lago-Rodríguez et al., 2014, for reviews on observational learning). This is because observation has much commonality with physical practice, which is the first determinant of motor skill learning. Specifically, it has been demonstrated that variables, such as the amount of practice (Carroll and Bandura, 1990; Blandin, 1994), the frequency of knowledge of results ([KR], Badets and Blandin, 2004, 2005; Badets et al., 2006), and the practice schedule (Blandin et al., 1994; Wright et al., 1997), affect learning via observational practice and physical practice in similar ways. These data led to the proposition that observation and physical practice use very similar processes. This proposition is supported by the results of neuroimaging studies that showed that an ensemble of neural structures (including the premotor cortex, the inferior parietal lobule, the superior temporal sulcus, the supplementary motor area, the cingulate gyrus, and the cerebellum), also called the “action observation network” (AON) (Kilner et al., 2009; Oosterhof et al., 2010), is activated both when individuals perform a given motor task and when they observe others performing that same motor task (Grafton et al., 1997; Buccino et al., 2001; Gallese et al., 2002; Cisek and Kalaska, 2004; Frey and Gerry, 2006; Cross et al., 2009; Dushanova and Donoghue, 2010; Rizzolatti and Fogassi, 2014; Rizzolatti et al., 2014).

Observation favors motor skill learning, but who should you observe to learn that new golf shot? An expert who masters the shot presumably will help you develop a reference of what to do and how to do it, but should you observe someone like you who is learning that shot and who presumably gives you a better chance of detecting and learning from errors or changes in strategy? Research has shown that observing both a skilled model (Martens et al., 1976; McCullagh et al., 1989; Lee et al., 1994; Al-Abood et al., 2001; Heyes and Foster, 2002; Hodges et al., 2003; Bird and Heyes, 2005) and a novice model leads to significant learning (Lee and White, 1990; McCullagh and Caird, 1990; Pollock and Lee, 1992; McCullagh and Meyer, 1997; Black and Wright, 2000; Buchanan et al., 2008; Buchanan and Dean, 2010; Hayes et al., 2010). However, recent results from our laboratory showed that observational learning of a new motor skill is improved following observation of both novice and expert models rather than either a novice or an expert model alone (Rohbanfard and Proteau, 2011; Andrieux and Proteau, 2013, 2014). We believe that this “variable” observation format leads to not only the development of a good movement representation (expert observation) but also the development of efficient processes for error detection and correction (novice observation).

In the present study, the question of interest is a simple but important one. When using a variable schedule of observation, will learning be better when the observers are informed beforehand of the “quality” of the performance they are about to see or will it be better when the observers are left to evaluate the performances before receiving feedback. Informing the observers of what they are about to see may enable them to select whether they will observe to imitate or rather observe to detect error, or weaknesses in the model's performance, which might facilitate the development of these processes. Alternatively, having the participants evaluate the performance quality they observed may activate more elaborate cognitive processes than when this information is fed forward (e.g., error detection and recognition, or evaluation of alternative strategy), thus resulting in better learning of the task.

The task that we chose required the participants to change the relative timing pattern that naturally emerged from the task constraints (Collier and Wright, 1995; Blandin et al., 1999) to a new, imposed pattern of relative timing. This is similar to changing one's tempo when executing a serve in tennis or a drive in golf (Rohbanfard and Proteau, 2011). The participants observed two models demonstrating a wide variety of performances. In one group, observers were informed before each trial of the quality level (expert, advanced, intermediate, novice, or beginner performance) of what they were about to see, whereas a second group of observers was provided the same information only after each observation trial was completed.

## Experiment 1

### Methods

#### Participants

Ninety right-handed students (45 males and 45 females; mean age = 20.5 years; *SD* = 0.9 years) from the Département de kinésiologie at the Université de Montréal participated in this experiment. The participants were naive to the purpose of the study and had no prior experience with the task, and all participants were self-declared as being right-handed. None of the participants reported neurological disorders, and all had normal or corrected-to-normal vision. The participants completed and signed individual consent forms before participation. The Health Sciences Research Ethics Committee of the Université de Montréal approved this experiment.

#### Apparatus and task

**Figure 1:**
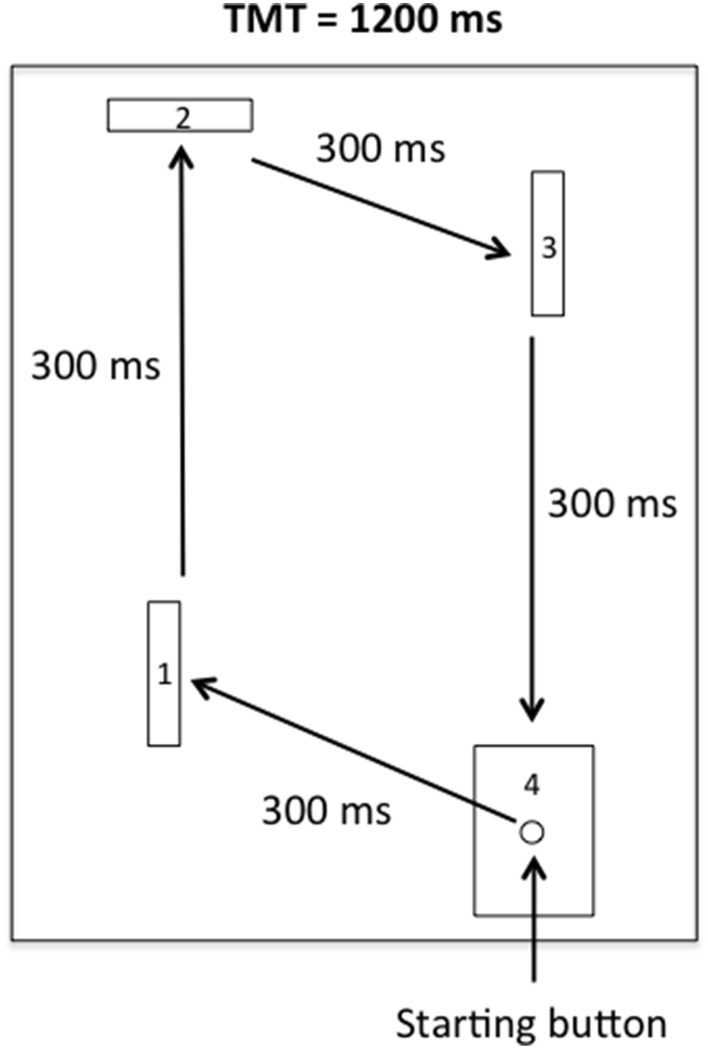
**Sketch of the apparatus**. Participants had to leave the starting button and hit the first, second, and third barriers in a clockwise motion before finally reaching the target.

The apparatus was similar to that used by Rohbanfard and Proteau (2011). As illustrated in Figure 1, it consisted of a wooden base (45 × 54 cm), three wooden barriers (11 × 8 cm), and a starting button embedded in a target (11 × 8 cm). The distance between the starting button and the first barrier was 15 cm. The distances of the remaining three segments of the task were 32, 18, and 29 cm, respectively. The barriers were placed perpendicular to the wooden base at the beginning of each trial, yielding a closed microswitch circuit. All of the microswitches were connected to a computer via the I/O port of an A–D converter (National Instruments, Austin, Texas, USA), and a millisecond timer was used to record both the total movement time (TMT) and the time required to complete each segment of the task (intermediate times, ITs).

For the physical practice trials (see below), the participants sat close to the starting position in front of the apparatus. Then, from the starting button, the participants were asked to successively knock down the first, second, and third barriers (thus releasing the microswitches) and finally hit the target in a clockwise motion as illustrated in Figure 1. Each segment of the task had to be completed in an IT of 300 ms, for a TMT of 1200 ms. The movement pattern, ITs, and TMT were illustrated on a poster located directly in front of the apparatus during all of the experimental phases.

#### Experimental phases and procedure

The participants were randomly assigned to one of the three groups, each consisting of 30 participants (15 females per group): control (C), feedforward KR and observation (FW), and observation and feedback KR (FB). All groups performed four experimental phases, spread over 2 successive days.

All participants received verbal instructions regarding TMT and ITs before the first experimental phase. The first experimental phase was a pre-test, in which all participants performed 20 physical practice trials without knowledge of the results (KR) on the TMT and the ITs.

The second phase was an acquisition phase and consisted of 60 observation trials for the participants in the two observation groups (FW and FB). These participants individually watched a video presentation of two models physically performing the experimental task. For each observation trial, KR concerning the model's performance (both TMT and ITs) was presented in ms (see Figure 1) either before the demonstration for the FW group or after the demonstration for the FB group. The model was changed every five trials (i.e., model 1: trials 1–5 and model 2: trials 6–10, and so on), for a total of 30 trials performed by one model and 30 trials performed by the other model. For both the FW and FB groups, the two models, who participated in previous work from our laboratory, were chosen because for both models, we had six video clips that illustrated performances in each one of five subcategories. Thus, the participants in the FW and FB groups could not associate one particular model with either a better or a poorer performance. An expert performance corresponded to a root mean square error (RMSE; see data analysis section for computation details) ranging between 0 and 15 ms; advanced, intermediate, novice, and beginner performances corresponded to RMSEs of 30–45 ms, 60–75 ms, 90–105 ms and 120+ ms, respectively. The participants in the FW and FB groups were informed of the model's performance in ms; they were also informed of the level of performance to which it referred. The resulting 30 trials of each model (five levels of performance × six repetitions) were randomized so that the five levels of performance were presented once into each set of five trials. To avoid physical imitation of the sequence, which could interfere with the observational processes, we asked the participants in the FW and FB groups to keep their hands on their thighs during the acquisition phase and to not reproduce the movements while watching the model(s). It was the Experimenter's main task to ensure that the participants complied with these instructions. The participants' overt behavior suggests that they did. Finally, participants of the control group did not physically practice or observe anything during this phase. Instead, they read a provided newspaper or magazine for the same duration as the observation for the other groups (approximately 10 min).

The third and fourth experimental phases were 10-min and 24-h retention phases. In each phase, all participants physically performed 20 trials with no KR. The participants were asked to complete each segment of the task in 300 ms, for a TMT of 1200 ms.

#### Data analysis

The data from the pre-test and the two retention phases were regrouped into blocks of five trials. For each successive block of five trials (i.e., trials 1–5, 6–10, etc.), we computed the absolute value of each participant's constant error (|CE|, the constant error indicates whether a participant undershot [negative value] or overshot [positive value] the total movement time) and variable error of the total movement time (VE or within-participant variability) to determine the accuracy and consistency of TMT, respectively. For intermediate times, we computed a RMSE, which indicates how much each participant deviated from the prescribed relative timing pattern in a single score. For each trial,

$$\begin{array}{l} \text{RMSE}=\sqrt{\sum \begin{array}{c} \text{Segment }4\\ \text{Segment }1 \end{array}\left(\frac{{\left(\text{ITi}-\text{target}\right)}^{2}}{4}\right)}, \end{array}$$

where ITi represents the intermediate time for segment “i,” and target represents the goal movement time for each segment of the task (i.e., 300 ms).

Because the data were not normally distributed (RMSE and time data are positively skewed), each dependent variable underwent a logarithmic transformation (ln). The transformed data for each dependent variable were independently submitted to an ANOVA contrasting three groups (C, FW, and FB) × three phases (pre-test, 10-min retention, 24-h retention) × four blocks of trials (1–5, 6–10, 11–15, and 16–20), with repeated measures on the last two factors. All of the significant main effects and simple main effects involving more than two means were broken down using Bonferroni's adjustment. For all comparisons, an effect was deemed significant if *p* < 0.05. Partial eta square ($\eta_{p}^{2}$) is the effect size reported for all significant effects (Cohen, 1988).

### Results

#### Total movement time

**Figure 2:**
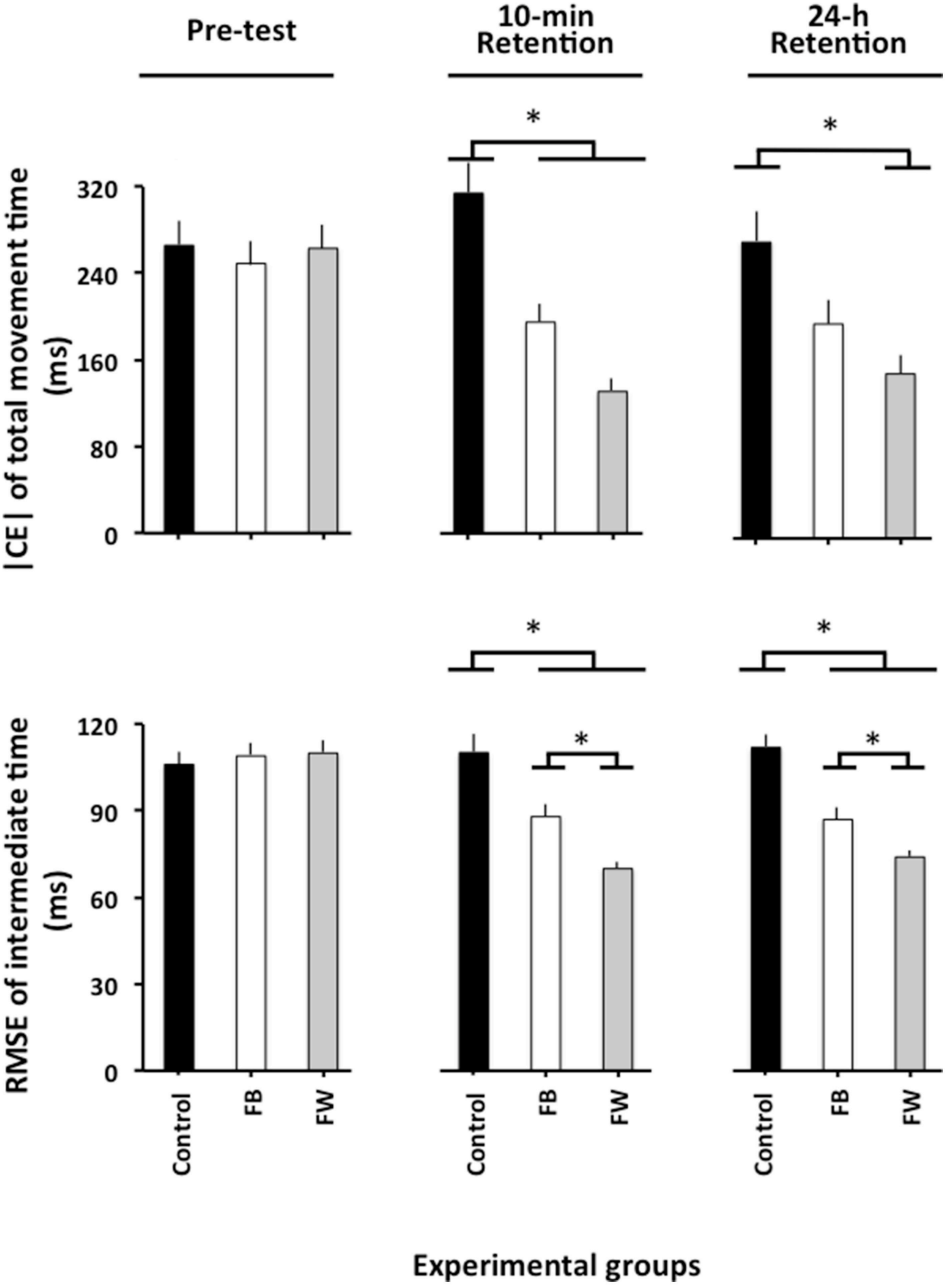
**Absolute constant error of TMT and root mean square error of relative timing as a function of the experimental phases and experimental groups (Experiment 1)**. ^*^p < 0.05. Error bars indicate standard error of the mean.

The ANOVA computed on |CE| (Figure 2, upper panel) revealed significant main effects for the variable group, *F*_(2, 87)_ = 5.04, *p* = 0.08, $\eta_{p}^{2}$ = 0.10, and phase, *F*_(2, 174)_ = 5.16, *p* = 0.007, $\eta_{p}^{2}$ = 0.06, as well as a significant phase × group interaction, *F*_(4, 174)_ = 4.93, *p* = 0.001, $\eta_{p}^{2}$ = 0.10. The breakdown of this interaction did not reveal any significant group differences in the pre-test (*F* < 1). In the 10-min retention test, *F*_(2, 87)_ = 10.12, *p* < 0.001, $\eta_{p}^{2}$ = 0.19, the *post-hoc* comparisons revealed that the control group had a significantly larger **|** CE**|** than both the FW and the FB groups (*p* < 0.05 in both cases), which did not differ significantly from one another (*p* = 0.19). In the 24-h retention test, *F*_(2, 87)_ = 4.34, *p* = 0.016, $\eta_{p}^{2}$ = 0.09, the FW group had a significantly smaller |CE| than the control group (*p* = 0.012)^1^.

The ANOVA computed on VE (not shown) revealed significant main effects for the variable phase, *F*_(2, 174)_ = 13.12, *p* < 0.001, $\eta_{p}^{2}$ = 0.13, and block, *F*_(3, 261)_ = 48.79, *p* < 0.001, $\eta_{p}^{2}$ = 0.36. *Post-hoc* comparisons of the phase effect revealed a larger VE of total time in the pre-test than in both the 10-min and the 24-h retention tests (*p* < 0.002 in both cases), which did not differ significantly from one another (*p* = 0.68). The block main effect resulted from a significantly larger VE of total time for the first than for the three remaining blocks of trials (*p* < 0.001 in all cases), which did not differ significantly from one another (*p*>0.05 in all cases).

### Relative timing

The ANOVA computed on the RMSE of relative timing revealed significant main effects for the variable group, *F*_(2, 87)_ = 21.49, *p* < 0.001, $\eta_{p}^{2}$ = 0.33, phase, *F*_(2, 174)_ = 39.98, *p* < 0.001, $\eta_{p}^{2}$ = 0.31 and block, *F*_(3, 261)_ = 14.77, *p* < 0.001, $\eta_{p}^{2}$ = 0.14, as well as a significant phase × group interaction, *F*_(4, 174)_ = 12.81, *p* < 0.001, $\eta_{p}^{2}$ = 0.23. The block main effect resulted from a significantly larger RMSE of relative timing for the first than for the three remaining blocks of trials (*p* < 0.001 in all cases), which did not differ significantly from one another (*p*>0.3 in all cases). More interestingly, the breakdown of the phase × group interaction (Figure 2, lower panel) did not reveal any significant group differences in the pre-test (*F* < 1). In the 10-min, *F*_(2, 87)_ = 14.85, *p* < 0.001, $\eta_{p}^{2}$ = 0.34, and the 24-h retention tests, *F*_(2, 87)_ = 23.23, *p* < 0.001, $\eta_{p}^{2}$ = 0.35, although the FB group significantly outperformed the control group (*p* = 0.001 in both cases), the FB group was, in turn, significantly outperformed by the FW group (*p* = 0.001 and *p* = 0.02, respectively)^2^.

### Discussion

The present experiment was designed to extend our knowledge of the observation conditions that optimize learning of a new relative timing pattern. In this learning situation, two observation groups, which observed a variety of demonstrations, were provided KR either before or after each trial during the acquisition phase. Specifically, we wanted to assess whether learning would be enhanced when the learners know the “quality” or characteristics of a demonstration before they observe the demonstration. The results are straightforward.

First, as illustrated in Figure 2, both the FW and the FB groups outperformed the control group on the retention tests. This was true for the learning of both the TMT and the relative timing. This expected result confirms previous findings that indicated that observation enables one to learn a new motor skill (see McCullagh et al., 1989; Hodges et al., 2007; Vogt and Thomaschke, 2007; Ste-Marie et al., 2012; Lago-Rodríguez et al., 2014, for reviews on observational learning) and, notably, a new relative timing pattern (Rohbanfard and Proteau, 2011; Andrieux and Proteau, 2013, 2014).

The most important finding of the present study is that the FB group was outperformed by the FW group in the retention tests. Although the two groups observed the same demonstrations, the results revealed that learning is optimized when one is given advance knowledge of the quality or characteristics of the witnessed demonstration. This finding fits well with previous reports from our laboratory (Rohbanfard and Proteau, 2011; Andrieux and Proteau, 2013) showing that a mixed observation regimen, in which the observers know who is the expert model and who is the novice model, favors learning of a new relative timing pattern better than either expert or novice observation alone.

Having advance knowledge that a less than perfect demonstration will be shown may be critical, considering that it has been reported that novice participants, such as in the present study, are not good at evaluating the quality of a demonstration. For example, Aglioti et al. (2008) had novice and expert basketball players observe video clips showing free-throw shots, and the video clips were stopped at different times before or immediately after the ball release. Expert basketball players and coaches/specialized journalists were better and quicker at predicting the fate of the shot (successful or not) than were novices (for similar results see also Wright et al., 2010; Abreu et al., 2012; Tomeo et al., 2013; Balser et al., 2014; Candidi et al., 2014; Renden et al., 2014).

The advantage of the FW over the FB protocol is important and, as far as we know, a similar finding has not been reported thus far. Therefore, a replication of this finding appeared important. In addition, we wondered whether the advantage noted for the FW protocol occurred only after a limited amount of observation. Finally, we were curious to see whether alternating the FW and the FB protocol would result in additive effects. We conducted Experiment 2 to address these questions.

## Experiment 2

### Methods

#### Participants

The 60 participants who volunteered for this experiment were drawn from the same population as that of Experiment 1 (36 males and 24 females; mean age = 22.7 years; *SD* = 4.9 years). The participants were naive concerning the purpose of this study and had no prior experience with the task. They completed and signed individual consent forms before participation. The Health Sciences Research Ethics Committee of the Université de Montréal approved this experiment.

#### Apparatus, task, experimental phases, procedure, and data analysis

We used the same task, apparatus, and procedures as in Experiment 1. The major difference between the present experiment and Experiment 1 is that participants performed two acquisition sessions, which led to a total of five experimental phases: pre-test, acquisition 1, immediate retention test, acquisition 2, and 24-h retention test.

The participants were randomly assigned to one of the three groups, each consisting of 20 participants (8 females per group): feedforward KR and observation during both acquisition 1 and 2 (FW1-2); feedforward observation and KR during acquisition 1 but observation and feedback KR during acquisition 2 (FW/FB); and observation and KR feedback during both acquisition 1 and 2 (FB1-2). We used the same video and models as in Experiment 1; however, the order of video presentation was different in acquisition 2 from that in acquisition 1. All participants were also informed that they would perform the same task after each acquisition phase, but with no KR concerning their own performance.

We used the same dependent variables and data transformation as in Experiment 1. For each dependent variable, we conducted a two-way ANOVA contrasting the three groups (FW1-2, FW/FB and FB1-2) × three experimental phases (pre-test, immediate retention, and 24-h retention). All of the significant main effects and simple main effects involving more than two means were broken down using Bonferroni's adjustment. For all comparisons, an effect was deemed significant if *p* < 0.05. Partial eta square ($\eta_{p}^{2}$) is the effect size reported for all significant effects (Cohen, 1988).

### Results

#### Total movement time

**Figure 3:**
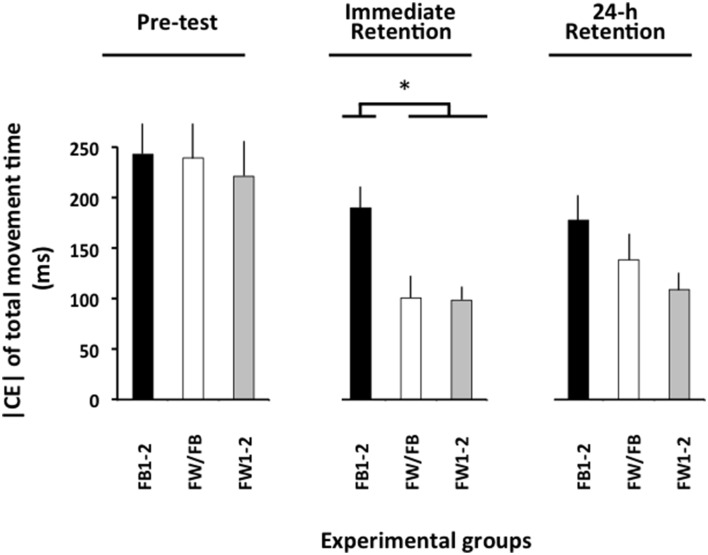
**Absolute constant error of TMT as a function of the experimental phases and experimental groups (Experiment 2)**. ^*^p < 0.05. Error bars indicate standard error of the mean.

The ANOVA computed for the |CE| of movement time (Figure 3) revealed significant main effects for the variable group, *F*_(2, 57)_ = 8.13, *p* = 0.001, $\eta_{p}^{2}$ = 0.22, and phase, *F*_(2, 114)_ = 21.13, *p* < 0.001, $\eta_{p}^{2}$ = 0.27, as well as a significant group × phase interaction, *F*_(4, 114)_ = 2.57, *p* = 0.042, $\eta_{p}^{2}$ = 0.08. The breakdown of this interaction did not reveal any significant group differences in the pre-test (*F* < 1). In the immediate retention test, *F*_(2, 57)_ = 10.27, *p* < 0.002, $\eta_{p}^{2}$ = 0.27, the FB1-2 group had a significantly larger **|** CE**|** than both the FW1-2 and the FW/FB groups (*p* < 0.001 in both cases), which did not differ significantly from one another (*p*>0.20). In the 24-h retention test, *F*_(2, 57)_ = 3.19, *p* = 0.049, $\eta_{p}^{2}$ = 0.10, the FW1-2 group had a slightly smaller |CE| than the FB1-2 group (*p* = 0.079)^3^.

The ANOVA computed on VE (not shown) revealed significant main effects for the variable group, *F*_(2, 57)_ = 7.82, *p* = 0.001, $\eta_{p}^{2}$ = 0.21, and phase, *F*_(2, 114)_ = 21.10, *p* < 0.001, $\eta_{p}^{2}$ = 0.27, as well as a significant group × phase interaction, *F*_(4, 114)_ = 4.38, *p* = 0.002, $\eta_{p}^{2}$ = 0.13. The breakdown of this interaction did not reveal any significant group differences in the pre-test (*F* < 1) and in the 24-h retention test, *F*_(2, 57)_ = 1.26, *p*>0.20. In the immediate retention test, *F*_(2, 57)_ = 10.26, *p* < 0.002, $\eta_{p}^{2}$ = 0.27, the FB1-2 group (62.7 ms) had a significantly larger VE than both the FW1-2 (51.1 ms) and the FW/FB (53.4 ms) groups (*p* < 0.001 in both cases), which did not differ significantly from one another (*p*>0.20)^4^.

#### Relative timing

**Figure 4:**
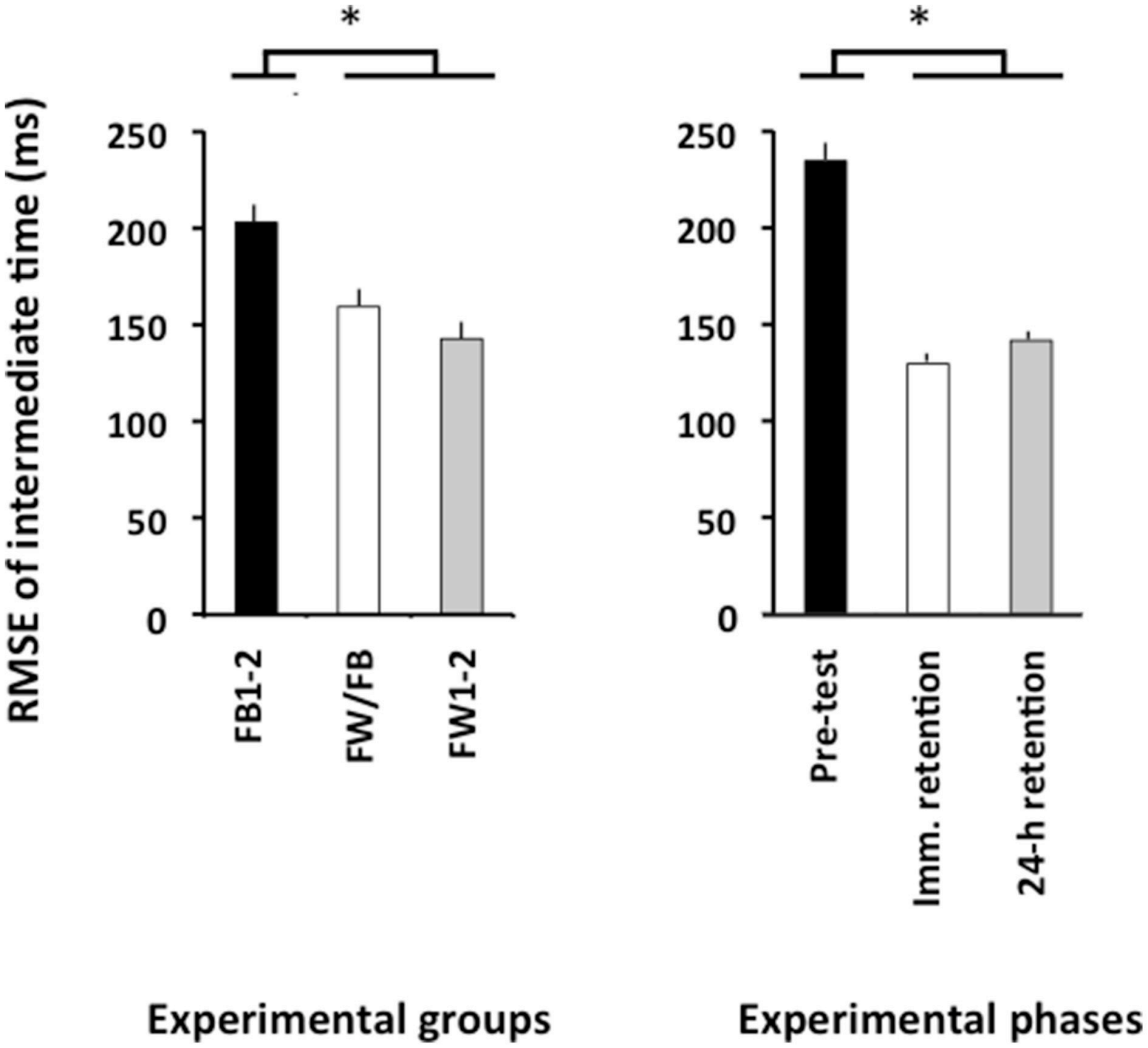
**Root mean square error of relative timing (Experiment 2) as a function of the experimental groups (left panel) and experimental phases (right panel)**. ^*^p < 0.05. Error bars indicate standard error of the mean.

The ANOVA computed for the RMSE of relative timing revealed significant main effects for the variable group, *F*_(2, 57)_ = 4.86, *p* = 0.01, $\eta_{p}^{2}$ = 0.15, and phase, *F*_(2, 114)_ = 78.21, *p* < 0.001, $\eta_{p}^{2}$ = 0.58. There was a significantly larger RMSE of relative timing in the pre-test than in both the immediate retention test and the 24-h retention test (*p* < 0.001 in both cases; see Figure 4, right panel), which did not differ significantly from one another (*p*>0.20). Finally, the FW1-2 and the FW/FB groups outperformed the FB1-2 group (*p* = 0.01 and *p* = 0.07; see Figure 4, left panel) but did not significantly differ from one another (*p*>0.20).

### Discussion

As expected, the decrease in error noted when going from pre-test to the retention tests supports previous findings indicating that observation aids learning of a new relative timing pattern (Blandin et al., 1999; Rohbanfard and Proteau, 2011; Andrieux and Proteau, 2013, 2014). More importantly, the results of Experiment 2 replicated those of Experiment 1, in that the FW1-2 group outperformed the FB1-2 group. Therefore, it can be safely concluded that learning to change the relative timing pattern that naturally emerges from the task's constraints to a new, imposed relative timing through observation is favored when one is informed of the model's performance prior to rather than after observation. Finally, the results also showed that what has been learned in a FB protocol does not add up to what can be learned in a FW protocol.

## General discussion

The main goal of the present study was to determine when in an observation protocol should KR concerning the model performance be provided, i.e., before or after each demonstration. The results of the two experiments of the present study clearly indicated that being informed of the model's performance before each demonstration favored learning of a new relative timing pattern better than when the observer was informed of the model's performance after each demonstration. Moreover, the results of Experiment 2 suggest that the advantage of the FW over the FB protocol remained significant even when the number of observation trials was doubled. Concerning this last point, we do not argue that a FW protocol should be favored in all cases and with all levels of expertise of the observers. Rather, we underline that the effect is reliable when novice observers are considered.

Our results may indicate that a feedforward observation protocol prepares the observer to engage specifically in either imitation processes when an expert or advanced performance is shown or in error detection processes when a beginner or novice performance is presented. This idea fits well with previous work from Decety et al. (1997), which stated that the patterns of brain activation during action observation depend on both the nature of the required executive processing and the extrinsic properties of the action presented. Specifically, these authors demonstrated that different areas of the brain become more active when one observes to recognize, which could be the case when observing a novice model or a poor or intermediate performance, and when one observes to imitate, which is likely to be the case when observing an expert model.

An alternative explanation of our findings could be that a FW protocol results in a “deactivation” of the AON when the participants were explicitly informed that a poor demonstration would follow. For instance, in an object-lifting task, it has been shown that the modulation of motor evoked potential (MEP) by transcranial magnetic stimulation (TMS) during observation of the lifting action is scaled to the force required to perform the grasping and lifting action (Alaerts et al., 2010a). It was also shown that when visual cues suggested that the object was heavier than in really was, the MEP modulation depended primarily on the observed kinematic profile rather than on the apparent weight of the object (Alaerts et al., 2010b; Senot et al., 2011). However, in a study by Senot et al. (2011), false explicit information concerning the weight of the object was provided in one experimental condition. This resulted in a conflict between the expected kinematic profile given the announced weight and the actual kinematic profile of the grasping and lifting action, leading to a “general inhibition of the corticospinal system.” Stated differently, at least a portion of the AON had been turned off. Therefore, it could be that the participants in our study turned off the AON when poor performance of the model was expected, leaving the AON active only for good trials.

This proposition is difficult to reconcile, however, with recent reports from our laboratory showing that observing both an expert and a novice model resulted in better learning of a new relative timing pattern than observing either a novice model or an expert model alone. If one could turn off the AON when informed that a poor demonstration will be shown (i.e., a novice model), then learning of the mixed observation group would have matched and not surpassed that of the expert observation group. Rather, going back to our first proposition, we suggest that a FW protocol helps novice performers detect and quantify errors in the model's performance, something they usually do poorly (Aglioti et al., 2008; Wright et al., 2010; Abreu et al., 2012; Tomeo et al., 2013; Balser et al., 2014; Candidi et al., 2014; Renden et al., 2014). In turn, the better detection and quantification of the model's performance may favor the development of inverse (Jordan, 1996) and forward models (Wolpert and Miall, 1996) of motor control.

In conclusion, observation is a powerful learning tool that is available to anyone and requires only minimal equipment to be used. It is now well-demonstrated that the benefits of observation for modifying the relative timing (i.e., tempo) of motor skill are enhanced when one has access to a variety of performances ranging from novices to experts either through variable or mixed observation schedules. The results of the present study suggest that those benefits are optimized if the observer knows beforehand the quality of the performance that she or he is about to observe during the first observation session. This could be very important in a classroom context in which a teacher/trainer would use a video observation protocol. For example, if the intention of the observer is to learn a specific aspect of a golf swing, it is likely that the result of the swing (i.e., the ball flight) will not be shown on the video. Therefore, the observer would not be able to “guess” the expertise of the model from the result of the swing and, as we have shown in the present study, learn better if he or she was informed in advance of the quality of what he or she is about to observe.

## Author contributions

All authors listed, have made substantial, direct and intellectual contribution to the work, and approved it for publication.

## Funding

This work was supported by a Discovery Grant (LP) provided by the Natural Sciences and Engineering Research Council of Canada (grant no. 111280-2013).

### Conflict of interest statement

The authors declare that the research was conducted in the absence of any commercial or financial relationships that could be construed as a potential conflict of interest.

## Footnotes

^1^To ascertain that the differences noted in the two retention tests between the control group and the FW and FB groups resulted from a significant decrease in the |CE| of total movement time, in a supplementary analysis we decomposed the group × phase interaction reported in the main text by computing a separate ANOVA for each group. The results revealed that for the control and the FB groups, the |CE| of total movement time did not significantly differ across the phases, [*F*_(2, 86)_ = 1.58, *p* = 0.21, $\eta_{p}^{2}$ = 0.04, and *F*_(2, 86)_ < 1, *p* = 0.37, $\eta_{p}^{2}=0.02$], respectively. On the contrary, for the FW groups, there was a significant main effect of the phases, *F*_(2, 86)_ = 11.60, *p* < 0.01, $\eta_{p}^{2}=0.1$, that revealed a significant decrease in the |CE| of total movement time going from the pre-test to the two retention tests (*p* < 0.01), which did not differ significantly from one another (*p*>0.10).

^2^As we did for the |CE| of total movement time, in a supplementary analysis we decomposed the group × phase interaction reported in the main text for the RMSE of relative timing by computing a separate ANOVA for each group. The results revealed that for the control group, the RMSE of relative timing did not significantly differ across the phases, *F*_(2, 86)_ = 0.32, *p* = 0.72, $\eta_{p}^{2}=0.01$. On the contrary, for both the FB and the FW groups, there was a significant main effect of the phases [FB: *F*_(2, 86)_ = 11.82, *p* < 0.001, $\eta_{p}^{2}=0.22$; FW: *F*_(2, 86)_ = 35.62, *p* < 0.001, $\eta_{p}^{2}=0.45$] that revealed a significant decrease in the RMSE of relative timing going from the pre-test to the two retention tests (*p* < 0.01), which did not differ significantly from one another (*p* > 0.10).

^3^As we did in Experiment 1, in a supplementary analysis we decomposed the group × phase interaction reported in the main text by computing a separate ANOVA for each group. The results revealed that for the FB1-2 group, the |CE| of total movement time did not significantly differ across the phases, *F*_(2, 56)_ < 1, *p* = 0.45, $\eta_{p}^{2}=0.03$. On the contrary, for both the FW1-2 and FW-FB groups, there was a significant main effect of the phases, [FW1-2: *F*_(2, 56)_ = 8.20, *p* = 0.001, $\eta_{p}^{2}=0.23$; FW-FB: *F*_(2, 56)_ = 13.76, *p* < 0.001, $\eta_{p}^{2}=0.33$] that, for both groups, revealed a significant decrease in the |CE| of total movement time going from the pre-test to the two retention tests (*p* < 0.01), which did not differ significantly from one another (*p*>0.10).

^4^For the VE of total movement time, the breakdown of the group × phase interaction revealed a significant main effect of phases for all three groups [FW1-2: *F*_(2, 56)_ = 10.27, *p* < 0.001, $\eta_{p}^{2}=0.27$; FW-FB: *F*_(2, 56)_ = 4.71, *p* = 0.013, $\eta_{p}^{2}=0.14$; FB1-2: *F*_(2, 56)_ = 7.38, *p* = 0.001, $\eta_{p}^{2}=0.21$]. For the FW1-2 and the FB1-2 groups, *pos-hoc* comparisons revealed a significantly larger VE in pre-test than in both retention tests (*p* < 0.01), which did not differ significantly from one another (*p*>0.30). For the FW-FB group, the VE of total movement time was significantly larger in the pre-test than in the 24-h retention test (*p* < 0.01).
